# Climate, Soil Management, and Cultivar Affect *Fusarium* Head Blight Incidence and Deoxynivalenol Accumulation in Durum Wheat of Southern Italy

**DOI:** 10.3389/fmicb.2016.01014

**Published:** 2016-06-30

**Authors:** Valeria Scala, Gabriella Aureli, Gaspare Cesarano, Guido Incerti, Corrado Fanelli, Felice Scala, Massimo Reverberi, Giuliano Bonanomi

**Affiliations:** ^1^Research Unit for Plant Pathology, Council for Agricultural Research and EconomicsRome, Italy; ^2^Research Unit for Cereal Quality, Council for Agricultural Research and EconomicsRome, Italy; ^3^Dipartimento di Agraria, University of Naples Federico IINaples, Italy; ^4^Plant Pathology, Dipartimento di Biologia Ambientale, Sapienza University of RomeRome, Italy

**Keywords:** FHB, deoxynivalenol, durum wheat, yield, environmental parameters, minimum tillage

## Abstract

*Fusarium* head blight (FHB) is a multifaceted disease caused by some species of *Fusarium* spp. A huge production of mycotoxins, mostly trichothecenes, often accompanied this disease. Amongst these toxic compounds, deoxynivalenol (DON) and its derivatives represent a major issue for human as well as for animal health and farming. Common and durum wheat are amongst the hosts of trichothecene-producing *Fusaria*. Differences in susceptibility to fungal infection and toxin accumulation occur in wheat cultivars. Recently, increasing incidence and severity of *Fusarium* infection and a higher DON accumulation in durum wheat were observed in Italy, especially in Northern regions. In this study, we analyzed wheat yield, technological parameters, the incidence of *Fusarium* infection and DON content in kernel samples of durum wheat coming from three locations of Southern Italy with different climatic conditions and grown during two seasons, with two methods of cultivation. Four different durum wheat cultivars prevalently cultivated in Southern Italian areas were chosen for this study. Our analysis showed the effects of environment and cultivar types on wheat productivity and key technological parameters for the quality level of the end-product, namely pasta. Notably, although a low rate of mycotoxin contamination in all study sites was assessed, an inverse relation emerged between fungal infection/DON production and durum wheat yield. Further, our study pinpoints the importance of environment conditions on several quality traits of durum wheat grown under Mediterranean climate. The environmental conditions at local level (microscale) and soil management practices may drive FHB outbreak and mycotoxin contamination even in growing area suitable for cropping this wheat species.

## Introduction

Durum wheat (*Triticum durum* Desf.) is the most widespread crop in the Mediterranean area. Sixty-seven percent of the Italian production of durum wheat comes from the Southern regions and it is used mainly for producing pasta (Fagnano et al., 2012). Quaranta et al. (2010) confirmed the importance of environmental local conditions in driving mycotoxin contamination in durum wheat. They reported that Southern Italy is an area particularly suitable for producing high quality durum wheat with a low content of *Fusarium*-toxins. Some species of *Fusarium* are the causal agents of the *Fusarium* Head Blight (FHB), a disease of great concern for wheat and for other cereal crops (Kelly et al., 2015). FHB disease causes direct economic losses including reduced yield and quality of grains and indirect loss due to mycotoxin contamination (Shephard, 2008; Berthiller et al., 2009; Zain, 2011). Climatic conditions, especially during wheat anthesis, consistently affect composition of *Fusarium* species causing FHB (Bernhoft et al., 2012; Covarelli et al., 2015; Kelly et al., 2015). *Fusarium* spp. associated to FHB disease (Xu et al., 2008) may change throughout the years (Covarelli et al., 2015), but *Fusarium graminearum* and *Fusarium culmorum* are the most commonly found species (Häller et al., 2008; Edwards, 2009). In Norway, Bernhoft et al. (2012) reported, “Agronomic and climatic factors explained 10–30% of the variation in *Fusarium* species and mycotoxins.” Since cereals from organic farming resulted less infected by *Fusarium* species than cereals from conventional farming systems, the authors conclude that this difference is mainly due to lack of crop rotation and use of mineral fertilizers and pesticides in conventional systems. In general, precipitation during anthesis is particularly conductive for cereal contaminations by *Fusarium* spp. (De Wolf et al., 2003; Fedak et al., 2007; Visconti and Pascale, 2010). The impact of FHB can be limited by adopting measures for reducing the inoculum and preventing its dispersal such as the cultural, biological, and chemical control and use of resistant varieties (Sutton, 1982; Magan et al., 2002). Czaban et al. (2015) suggested that winter wheat kernel infection by *Fusarium* spp. depends primarily by weather conditions and then by the wheat genotype.

Previous crop residue and tillage practices differentially affected the incidence and severity of FHB disease (Dill-Macky and Jones, 2000). Schaafsma et al. (2005) confirmed that planting wheat after corn or wheat, together with minimal or no-tillage practices increased the potential for FHB epidemics across South-Western Ontario. Miller et al. (1998) also reported that zero tillage resulted in increased seed infection compared to conventional tillage in Canada. Further, data concerning the influence of environment on several technological parameters of durum wheat kernels are of great interest (Flagella et al., 2010; Pinheiro et al., 2013). In Italy, FHB has been reported mostly in the Northern–Central regions. Disease incidence and *Fusarium* species involved, varied depending on the year, cultivation area, and wheat cultivar. Infections increase gradually from the South to the North and are closely related to the amount of precipitations during wheat anthesis (Pancaldi et al., 2010; Covarelli et al., 2015).

Several aspects concerning the effect on quality traits of durum wheat by species of fungi involved in FHB disease are already assayed, as for damage on protein fractions of kernels (Dexter et al., 1997; Nightingale et al., 1999; Brzozowski et al., 2008). Other than direct damaging effects on kernels, FHB may produce different types of trichothecenes: *Fusarium* spp. producing DON and/or its acetylated derivatives are described as chemotype I, whereas those produce nivalenol (NIV) and/or 4-acetyl-NIV are included in chemotype II (Pasquali and Migheli, 2014). DON is the most frequent *Fusarium*-toxin in Italy, as well as in other European countries. Its occurrence in durum wheat increases from Southern to Northern areas in Italy, with a heavy influence of some factors such as year and area of cultivation (Aureli et al., 2015). DON is toxic for humans, animals, and contributes to the aggressiveness of *F. graminearum* during wheat infection. Resistance to DON is an important aspect of wheat resistance to FHB (Rocha et al., 2005; Gauthier et al., 2013; Pasquali and Migheli, 2014). Since durum wheat is more susceptible to FHB than common wheat (Covarelli et al., 2015) mycotoxin accumulation in kernels is of particular concern in Italy as food safety issue (Boutigny et al., 2008; Covarelli et al., 2015). The European Commission established maximum thresholds (EU Commission Regulation No. 1881/2006 and 1126/2007) and “indicative levels” (Recommendation 2013/165/EU) for the T-2 and HT-2 *Fusarium*-toxins content in cereals and cereal based products.

In this paper, we study the influence of climatic conditions on durum wheat grown in cropping trials performed in Southern Italy that is known as a suitable growing area for growing this species of wheat. Several quality traits, infection by trichothecene-producing *Fusarium* spp. and accumulation of deoxynivalenol (DON) in grains were assessed in four cultivars of durum wheat grown with two techniques in three locations during two growing seasons.

## Materials and methods

### Study sites description

Experiments were conducted at three study sites in Southern Italy in the area of Lacedonia (Avellino) included in the Campania region (Figure S1) during two growing seasons (2011–2012 and 2012–2013). The three sites are characterized by different microclimatic conditions, related to altitude and aspects, but similar soil types (Calcixerert Vertisols; Soil Survey Staff, 1998). Soil characteristics including pH, organic C, total N, available P_2_O_5_, K^+^, and electrical conductivity were rather similar at the three sites (Table S1). This selection of study sites allows an evaluation of the microclimate impact on wheat production and disease incidence by keeping constant soil type as ecological factor. All sites share a Mediterranean climate, with differences related to altitude and aspect. Site A is located at 827 m a.s.l. (41°02′00.99″N, 15°27′10.82″E) on an almost flat hilltop. Site B is at 520 m a.s.l. (41°01′20.65″N, 15°30′05.55″E) with a western face aspect, while site C is at 513 m a.s.l. (41°03′16.98″N, 15°30′32.45″E) with East exposure. Mean annual air temperature is 13.1, 13.6, and 14.5°C and the mean annual rainfall is 512, 510, and 523 mm at study sites A, B, and C, respectively (WorldClim 1.4.; Hijmans et al., 2005). To assess microclimate conditions at the three sites we installed monitoring meteorological stations (Vantage Pro2 Plus, Davis, USA), each of which was equipped with a data logger and integrated sensors to collect the following hourly weather data: air temperature, air relative humidity and rainfall (Figure S2).

### Experimental design, crop management, and sampling

Each experimental trial was carried out according to a randomized complete block experimental design with three replications totalling 24 permanent plots (50 × 25 m) were established at each site; this experimental protocol was the same both for year and within each site (A, B, and C) but not overlapping the plots areas sowed in the previous year. Two tillage treatments: (i) conventional tillage (CT), consisting of mouldboard plowing to 40 cm depth followed by a soil grubber and a disk harrow passage for seedbed preparation, and (ii) minimum-tillage (MT) consisting of a single passage by disk harrow to a depth of 8–10 cm for seedbed preparation. Mouldboard plowing was used because is still the most common tillage in the study area. On both CT and MT soils the following durum wheat cultivars with different growth cycle length, from early (E) to medium (M) were sown: Svevo (E), Simeto (ME), Claudio (M), and Normanno (M). The four cultivars employed in this assay were chosen based on their large diffusion in the area considered and the different length of the growth cycle. The sowing period ranged from 15^th^ to 17^th^ Nov. 2011 and from 11^th^ to 27^th^ Nov. 2012; the harvesting period ranged from 23^th^ Jun. to 11^th^ Jul. 2012 and from 6^th^ to 25^th^ Jul. 2013. Crop management was carried out according to local agronomic professional practice. The seeding rate used was of 400 seeds/mq/plot. Previous crop rotation at all sites was wheat-faba bean (*Vicia faba minor*). Based on soil analyses (Table S1), mineral nitrogen (120 kg N ha^−1^) was split applied, at the rate of 1/3 before sowing and 2/3 N top-dressed applied during wheat tillering as ammonium nitrate. Weeds were periodically controlled during the growing season by means of specific and selective herbicides. At harvest time, approximately 30 kg grain samples grains were random collected from each plot directly from the threshing machine. After homogenization, subsample of 5 kg was taken from which subsequent subsample of 1 kg was taken again. A final grain sample of 100 g was milled for the analyses.

All sampling operations were based substantially on the criteria of representativeness reported by European legislation (EU Commission Regulation No. 401/2006).

### Analysis of quality parameters

The semolina samples obtained by a pilot milling plant (Buhler MLU 202) were employed for the following quality analyses: protein content carried out by Dumas combustion method (ICC method n. 167) with automatic instrument Leco FP 428 (USA), gluten content (EN ISO 21415), gluten index (ICC 158), Glutomatic System (Perten, Sweden), rheological parameters (alveographic P/L and alveographic W; alveograph Chopin—UNI 10453 method), yellow and brown indices by reflection colorimeter (Minolta Chromameter CR-400). Pasta samples (spaghetti shape, 1.65 mm diameter) were produced by an experimental press (Namad, Italy) and by an experimental drying system (AFREM-France) at low-temperature (50°C) drying diagram. The overall judgment was carried out evaluated by a score ranging from 10 to 100 (D'Egidio et al., 1993). The results showed are the average values of replicate analyses as specified for each method employed.

### Fungal identification and infection incidence

Fifty kernels were randomly selected from each sample and surface disinfected accordingly to Giorni et al. (2015). Grain kernels were plated on Petri dishes (Ø 9 cm) containing Potato Dextrose Agar (PDA, Oxoid Ltd., Basingstoke, Hampshire, UK) added with 0.1% streptomycin (Sigma-Aldrich, St. Louis, MO, USA) and incubated at 25°C for 7 days with a 12 h light photoperiod. After incubation, kernels showing mold development were counted and incidence percentage calculated as in Giorni et al. (2015). *Fusarium, Aspergillus*, and *Penicillium* growing colonies were identified at genus level (Raper and Fennell, 1965; Pitt, 1979; Summerell et al., 2003). All *Fusarium* isolates were sub-cultured on water agar (2% of Bacto agar, Difco) using the single spore technique. The *Fusarium* spp. mycelia used for the DNA extraction were grown on PDA. Glumes and spikelets were evaluated for FHB severity at harvest. FHB severity was estimated by counting infected spikelets in a head and expressed as percentages (Burlakoti et al., 2007). FHB severity values were calculated from 90 wheat heads (30 heads per replicate and a total of 90 heads) per treatment derived from the experimental design previously described.

### Fungal growth by qPCR

Total DNA was extracted from wheat kernels as described in Reverberi et al. (2013) and its concentration and quality was determined using by spectrophotometer (DU-530, Beckman Instrument Inc.). The total DNA extract from kernels was used as template to monitor *Fusarium* trichothecene-producer (TR-producing *Fusarium*) growth in durum wheat kernels. At this aim, a specific SYBR green qPCR method was set by using *Tri5* primers (for_CAGATGGAGAACTGATGGT; rev_GCACAAGTGCCACGTGAC) as described by Edwards et al. (2001). *Tri5* primers yielded a 260-bp fragment. Standard calibration was performed plotting the Real-time PCR signals obtained for genomic DNA of *Fusarium* spp. mycelia, harvested from 7-day-old single-spore cultures (see description in chapter 2.4), in the concentration range 0.01 pg–100 ng. The equation, describing the increase of *Fusarium* trichothecene-producer (TR-producing *Fusarium*) DNA concentration, was calculated (*y* = −0, 9829*x*+27,921, *R*^2^ = 0.994) and used for extrapolating quantitative information of DNA targets of unknown concentrations in wheat kernels. The efficiency of the PCR reaction (98.8%) was obtained from the calibration curve slope (*E* = 10^−1^/slope^−1^). In all samples, DNA was extracted in triplicate and each biological replicate was technical repeated three times.

### Deoxynivalenol analysis

From each grain sample a representative subsample was milled (particle size ≤ 0.5 mm) by the use of Cyclotec 1093 (FOSS, Sweden) and submitted to the DON analyses by Enzyme-Linked Immuno-Sorbent Assay (ELISA). DON determination was made according to the Ridascreen® DON method (R-Biopharm AG, Germany). The limit of detection (LOD) was 18.5 μg/kg. The range of recovery declared in the method was between 85 and 110%. Data were acquired on such as samples as mean of double analysis (CV ≤ 10%). The Basic Robotic Immunoassay Operator (BRIO, SEAC, Radim Group, Italy) was used and the absorbance values were read using Sirio-S Microplate Reader (SEAC, Radim Group, Italy). The RIDA® Soft Win software (R-Biopharm AG, Germany) was employed for quantitation of DON in samples. Distilled water was obtained from Water Purification System Zeener Power I (Human Corporation, Korea).

### Statistics

We considered the following 11 dependent variables: content of DNA from TR-producing *Fusarium* and of deoxynivalenol toxin (DON) in wheat kernels, yield (total wheat production), percent content of proteins and gluten, gluten index, alveographic W and P/L ratio, yellow and brown indices, and sensorial assessment score, assessed in wheat samples undergoing 48 different combinations of cultivation treatments. First, a cross-correlation matrix was calculated among the dependent variables. Then, for each dependent variable, a combined analysis by Generalized Linear Model (GLM) was fitted, including main and second order interactive effects of treatments: wheat cultivar (four levels: Claudio, Normanno, Simeto, Svevo), soil management practice (either conventional tillage, CT or minimum tillage, MT). Experimental replication factors including harvesting year (either 2012 or 2013) and experimental field (three sites: A, B, and C) were considered as additional treatments in the GLMs after preliminary verification, for each dependent variable, of the homogeneity of variances for different years and for different sites. In the case of harvesting years (*N* = 2) the homogeneity of variances were tested by the *F*-test, while in the case of sites (*N* = 3) the Bartlett's chi-square test was used. In the GLMs, pairwise significant differences between treatment combinations were assessed by Duncan's *post-hoc* test. Statistical significance was tested at the conventional threshold of α = 0.05. The data matrix of dependent variables and treatments was submitted to Principal Component Analysis. Loading vectors of variables and factorial scores of treatment combinations were plotted in 3D biplots representing the first three principal components (Jolliffe, 2002). All statistical analyses were carried out using the software package STATISTICA 7 (StatSoft Inc., Tulsa, Oklahoma).

## Results

### Quality parameters assessment

**Figure 1:**
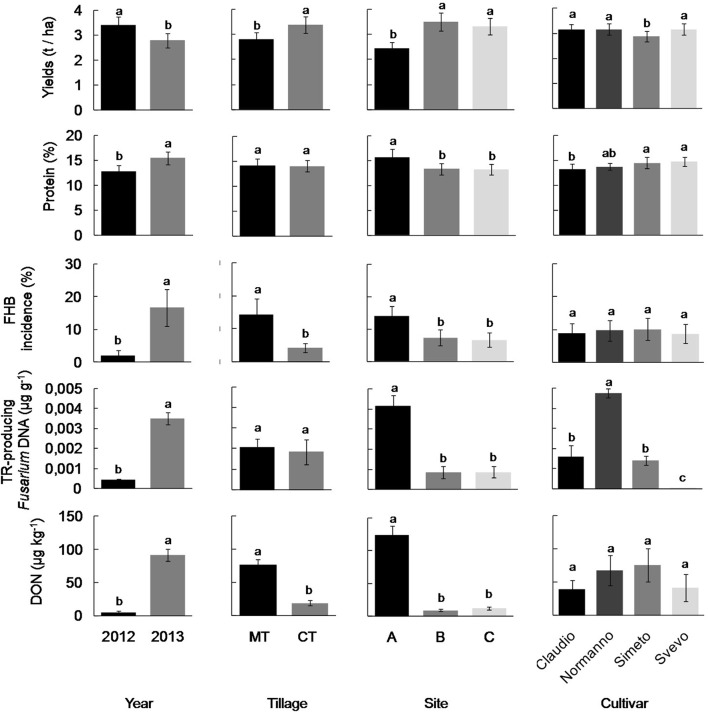
**Grain yield, FHB incidence, and content of proteins, DNA of trichothecene-producing *Fusarium* spp. and deoxynivalenol toxin (DON) in wheat kernels in different years (2012 and 2013), soil management regimes (conventional tillage—CT vs. minimum tillage—MT), study sites along a decreasing rainfall gradient (sites A, B, and C), and different cultivars (Claudio, Normanno, Simeto, Svevo)**,. For each bar, data refer to mean and standard deviation. Different letters indicate statistically significant differences within each plot (Duncan test, *P* < 0.05, for statistical detail see Table 1).

Results of GLM and *post-hoc* Duncan tests showed that crop yields were significantly affected by all experimental factors, with also significant interactions of wheat cultivar with cultivation year and study site (Table 1; Table S2; Supplementary Datasheet 1). In detail, yield was significantly higher in 2012 compared to 2013, in sites B and C compared to site A and lower for the cultivar Simeto (Figure 1); concerning soil tillage regime, wheat yield was significantly higher in CT compared to MT (Figure 1).

**Table 1:** **Results of Generalized Linear Models (GLMs) for 11 dependent variables assessed in 48 samples of durum wheat undergoing different cultivation treatments, including harvesting year (Y, either 2012 or 2013), wheat cultivar (Cv, four levels), experimental field (S, three sites) and soil management practice (M, either conventional tillage or minimum tillage)**.

**Effect**	***d.f*.**	***SS***	***MS***	***F***	***p***	***SS***	***MS***	***F***	***p***
		TR-producing *Fusarium* DNA in kernels (ng g^−1^)^*^				**Yield (t ha^−1^)**			
Year (Y)	1	1.1364	1.1364	22.401	<*0.0001*	439.23	439.23	80.872	<*0.0001*
Cultivar (Cv)	3	1.4373	0.4791	9.444	*0.0003*	63.80	21.27	3.915	*0.0214*
Study site (S)	2	1.1671	0.5836	11.503	*0.0003*	1026.25	513.12	94.478	<*0.0001*
Management (M)	1	0.0069	0.0069	0.136	0.7160	406.00	406.00	74.754	<*0.0001*
Y × Cv	3	1.1493	0.3831	7.552	*0.0011*	248.04	82.68	15.223	<*0.0001*
Y × S	2	1.4878	0.7439	14.664	<*0.0001*	1.82	0.91	0.168	0.8468
Cv × S	6	1.0054	0.1676	3.303	*0.0171*	431.59	71.93	13.244	<*0.0001*
Y × M	1	0.0003	0.0003	0.005	0.9428	1.47	1.47	0.271	0.6079
Cv × M	3	0.0211	0.0070	0.139	0.9358	6.28	2.09	0.385	0.7646
S × M	2	0.0068	0.0034	0.067	0.9357	7.09	3.54	0.652	0.5302
		**Deoxynivalenol (DON, μg/kg)**				**Overall judgment score**			
Year (Y)	1	89096	89096	8.9890	*0.0064*	0.59	0.59	0.025	0.8749
Cultivar (Cv)	3	31115	10372	1.0464	0.3909	47.83	15.94	0.682	0.5719
Study site (S)	2	136721	68361	6.8969	*0.0045*	23.39	11.70	0.501	0.6127
Management (M)	1	40021	40021	4.0377	0.0564	25.037	25.037	1.071	0.3114
Y × Cv	3	32088	10696	1.0791	0.3775	59.94	19.98	0.855	0.4783
Y × S	2	134167	67084	6.7681	*0.0049*	42.78	21.39	0.915	0.4145
Cv × S	6	37806	6301	0.6357	0.7005	496.01	82.67	3.537	*0.0125*
Y × M	1	41184	41184	4.1551	0.0532	10.70	10.70	0.458	0.5053
Cv × M	3	18307	6102	0.6157	0.6118	21.24	7.08	0.303	0.8229
S × M	2	71304	35652	3.5970	*0.0437*	49.59	24.79	1.061	0.3625
		**Proteins (% DW)**				**Gluten (% DW)**			
Year (Y)	1	87.50	87.50	143.045	<*0.0001*	27.183	27.183	59.3236	<*0.0001*
Cultivar (Cv)	3	17.60	5.87	9.590	*0.0003*	22.573	7.524	16.4211	<*0.0001*
Study site (S)	2	63.31	31.65	51.750	<*0.0001*	21.428	10.714	23.3821	<*0.0001*
Management (M)	1	0.06	0.06	0.092	0.7650	1.066	1.066	2.3271	0.1408
Y × Cv	3	1.75	0.58	0.954	0.4309	0.581	0.194	0.4225	0.7386
Y × S	2	3.88	1.94	3.174	0.0606	2.838	1.419	3.0965	0.0644
Cv × S	6	6.17	1.03	1.682	0.1705	5.212	0.869	1.8956	0.1248
Y × M	1	0.42	0.42	0.679	0.4183	0.098	0.098	0.2138	0.6481
Cv × M	3	2.69	0.90	1.465	0.2502	1.406	0.469	1.0227	0.4008
S × M	2	0.19	0.09	0.155	0.8577	1.285	0.643	1.4027	0.2662
		**Gluten index**				**Alveographic W (J × 10^−4^)**			
Year (Y)	1	0.6	0.6	0.008	0.9306	81263	81263	61.147	<*0.0001*
Cultivar (Cv)	3	3074.3	1024.8	12.874	<*0.0001*	9031	3010	2.265	0.1079
Study site (S)	2	631.0	315.5	3.964	*0.0332*	42033	21017	15.814	<*0.0001*
Management (M)	1	28.7	28.7	0.361	0.5538	3153	3153	2.372	0.1372
Y × Cv	3	52.3	17.4	0.219	0.8822	5675	1892	1.423	0.2615
Y × S	2	118.4	59.2	0.744	0.4863	15953	7976	6.002	*0.0080*
Cv × S	6	1112.3	185.4	2.329	0.0665	41127	6854	5.158	*0.0017*
Y × M	1	65.0	65.0	0.817	0.3755	463	463	0.348	0.5610
Cv × M	3	35.0	11.7	0.147	0.9307	2133	711	0.535	0.6629
S × M	2	110.8	55.4	0.696	0.5088	158	79	0.059	0.9425
		**Alveographic P/L ratio**				**Yellow index**			
Year (Y)	1	24.496	24.496	17.474	*0.0004*	74.63	74.63	58.130	<*0.0001*
Cultivar (Cv)	3	32.041	10.680	7.619	*0.0010*	185.64	61.88	48.201	<*0.0001*
Study site (S)	2	32.798	16.399	11.698	*0.0003*	5.66	2.83	2.203	0.1333
Management (M)	1	0.045	0.045	0.032	0.8593	0.001	0.001	0.001	0.9809
Y × Cv	3	2.029	0.676	0.483	0.6976	10.84	3.61	2.814	0.0618
Y × S	2	45.266	22.633	16.145	<*0.0001*	1.22	0.61	0.474	0.6287
Cv × S	6	6.602	1.100	0.785	0.5906	12.79	2.13	1.661	0.1759
Y × M	1	0.006	0.006	0.004	0.9490	0.19	0.19	0.147	0.7049
Cv × M	3	3.980	1.327	0.946	0.4346	1.24	0.41	0.321	0.8100
S × M	2	0.016	0.008	0.006	0.9944	2.65	1.32	1.031	0.3725
		**Brown index**							
Year (Y)	1	9.937	9.937	49.379	<*0.0001*				
Cultivar (Cv)	3	10.014	3.338	16.586	<*0.0001*				
Study site (S)	2	7.870	3.935	19.554	<*0.0001*				
Management (M)	1	1.216	1.216	6.043	*0.0219*				
Y × Cv	3	0.411	0.137	0.681	0.5725				
Y × S	2	2.455	1.228	6.101	*0.0075*				
Cv × S	6	3.005	0.501	2.488	0.0529				
Y × M	1	0.065	0.065	0.321	0.5767				
Cv × M	3	0.463	0.154	0.767	0.5242				
S × M	2	0.257	0.129	0.639	0.5371				

*df, degrees of freedom; SS, sum of squares; MS, mean square; F and p, statistics for main and second order interactive effects of treatments*.

* *For best visualization, SS and MS of TR-producing Fusarium DNA data have been multiplied by 10^10^ for content in kernels*.

The protein and gluten content were significantly affected by all experimental factors with the exception of soil management whereas the cultivar factor had the major influence on gluten index parameter (Table 1). Protein content was higher in 2013, at site A, and was significantly lower for cultivar Claudio compared to Simeto and Svevo (Figure 1). The year and the study site factors had a clear and significant influence on the alveographic parameters W and P/L ratio, while their interaction was significant only for P/L parameter.

The environmental factors influenced the brown index, a negative characteristic of semolina that is also influenced by the mineral content of grain, whereas only cultivar and year factors affected the yellow index color. With regard to this last character, the results obtained indicate the significant influence of genotype and environmental factors on grain quality parameters. Finally, the overall judgment score was not affected by any of the experimental factors (Table 1; Table S2).

### *Fusarium* spp. infection and DON accumulation

The kernels sampled in 2012 and 2013 showed a different incidence of total fungal infection, with *Fusarium* spp. as the main fungal contaminants in 2013 (Table S3). GLM analysis showed that the content of TR-producing *Fusarium* DNA in kernels was significantly affected by all experimental factors, with the exception of soil tillage regime; the year and its interaction with the study site and cultivar type had the major significant influence (Table 1). In particular, fungal DNA content as well as FHB severity was higher in 2013 compared to 2012 with a major presence of fungal DNA content in grain samples of cultivar Normanno, especially at the coldest and wettest study site (A; Figure 1). Deoxynivalenol accumulation in wheat kernels was significantly affected by year, study site and by their interactions but not by the cultivar type (Table 1). DON reached higher concentrations in 2013 compared to 2012 at the site (A; Figure 1). The sole soil tillage regime partly (*p* = 0.056) affected DON content in kernel, whereas its interaction with the study site significantly influences the toxin content, with higher values of concentration in MT compared to CT (Figure 1).

### Relationship among fungal infection, DON contamination and grain quality parameters

**Figure 2:**
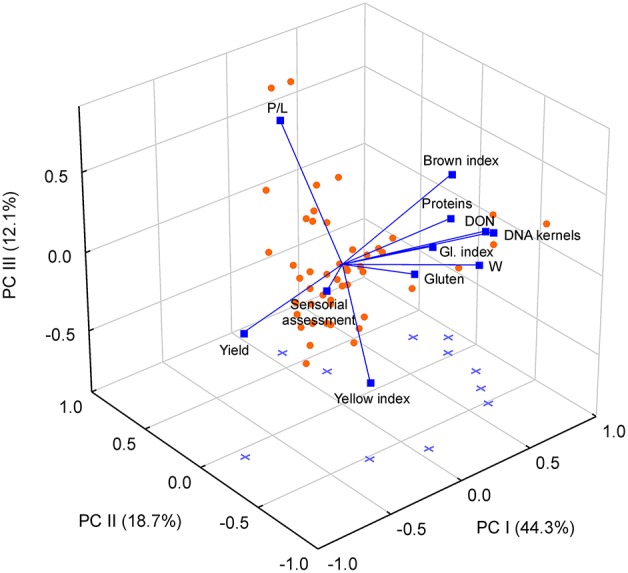
**Biplot from Principal Component Analysis (PCA) of 11 durum wheat variables (total wheat production, content of proteins, gluten, DNA of trichothecene-producing *Fusarium* in kernels, deoxynivalenol toxin (DON), gluten index, alveographic W, alveographic P/L ratio, yellow and brown indices, and overall judgment of pasta) assessed in 48 samples differing by wheat cultivar and cultivation year, site and type of soil management**. Data refer to loading vectors of variables (blue vectors) and factorial scores of samples (orange dots).

The multivariate PCA ordination of dependent variables and treatments showed a positive association of the first principal component with most of the grain quality parameters (Figure 2). The second principal component was positively associated with alveographic P/L ratio and negatively with yellow index and gluten index (Figure 2), while the third ordination axis was positively related with P/L and negatively with overall sensorial assessment score.

Yield was the only variable negatively associated with the first principal component, indicating that this parameter was inversely proportional to some indicators of high-quality (e.g., protein and gluten content and W), as well as to other ones related to low-quality (i.e., brown index, Table S4). The negative correlation between protein content and yield parameters was in agreement with previous data (Mangini, 2006; Blanco et al., 2011). Yield, being unrelated to the second and third components, was not associated with other quality indices (i.e., gluten index, P/L, yellow index, sensorial assessment score, Table S4). DON content and fungal infection parameters (content of TR-producing *Fusarium* DNA in kernels) were both positively correlated with the first principal component, indicating a negative effect of fungal infection on yield even with low levels of contamination.

### Weather conditions

Weather conditions during wheat flowering, a process occurring in May at our study sites, are the key factor for *Fusarium* infection and disease spread (De Wolf et al., 2003). Air temperature and RH as well as daily rainfall in May 2012 and 2013 are summarized in Figures S2–S4. In 2012, the anthesis stage occurred between 20^th^ and 25^th^ (site A), 11^th^ and 15^th^ (site B), and 10^th^ and 14^th^ day (site C) of May whereas in 2013 between the 28^th^ of May and 2^th^ of June (site A), 10^th^ and 16^th^ of May (site B), 14^th^ and 19^th^ of May (site C). Temperature and rainfall were consistently different between the two years and among study sites. Site A, located at the highest elevation, was the coldest with relatively high humidity levels in both years (Figure S2). Mean monthly temperature in 2012 was consistently lower than in 2013 at all study sites (Figure S2). In addition, lower cumulated rainfall, fewer rain events along the flowering periods, and higher air relative humidity were recorded in 2012 compared to 2013 (Figures S2, S3). Some of the rainy events recorded in 2012 and 2013 were followed by several days of dry weather, with low air relative humidity (Figures S2, S3). However, the fewer rain events recorded at site A in 2013 from the 25th day to the end of the month were followed by higher air relative humidity as well as lower temperature trend compared to 2012: these are to the most suitable conditions for FHB spread during the anthesis phase.

## Discussion

Durum wheat is the most widespread crop in the Mediterranean area. Our study suggests that environmental conditions at local level (microscale) and soil management practices are determinant factors in controlling potential FHB outbreak and mycotoxin contamination. This study, based on a two-year field experiment at three study sites of Southern Italy, confirms the critical role of weather conditions in promoting the development of *Fusarium* species, producers of trichothecenes, even in areas suitable for cropping durum wheat. Intriguingly, it appears that data related to different climates such as those in USA (De Wolf et al., 2003), Canada (Hooker et al., 2002), and Northern Europe (Chandelier et al., 2011; West et al., 2012) can be applied to Mediterranean conditions of Southern Italy. Previous crop residues and tillage practices can also affect incidence and severity of FHB (Dill-Macky and Jones, 2000). In Italy, FHB as well as DON contamination have been reported in several regions with different intensity depending on the year, cultivation area and durum wheat variety (Pancaldi et al., 2010; Aureli et al., 2015). Infection rates increase with a South-North gradient and closely relate to precipitations during wheat anthesis (Pancaldi et al., 2010; Covarelli et al., 2015).

Our study, reporting the highest presence of TR-producing *Fusaria* and DON contamination in the wettest study site (site A) and year (2013). These results *de facto* extend to Southern Italy area, previous data obtained in other studies. In fact, FHB incidence and the amount of DNA of trichothecene-producing *Fusarium* into durum wheat kernels significantly varied between the two observation periods, being higher in 2013 than in 2012 as well as the DON content in kernels. Apparently, the two different soil management regimes had no influence on the fungal DNA amount in the wheat kernel, whereas the climatic features of the cultivation areas consistently affected it. In the site A, the highest values of cumulated rainfall and mean air humidity were recorded and associated with the highest values of fungal DNA content and DON contamination in wheat kernels. Besides environmental conditions, the wheat cultivar influenced fungal infection and the cultivar Normanno showed the higher content of TR-producing *Fusarium* DNA (Figure 1).

DON is the most frequent *Fusarium*-mycotoxin detected in Italy, as well as in other European countries; nevertheless, FHB-associated species of *Fusarium* can produce different types of trichothecenes (Pasquali and Migheli, 2014). DON occurrence in our grain samples, showed very low levels of contamination (maximum: 734 μg/kg), even if a slight difference between 2012 and 2013 harvest was measured (Figure 1). Incidence of positive samples (DON concentration ≥18.5 μg/kg) on the total samples analyzed was 21% in 2012 and 71% in 2013 whereas the average of contamination values of the positive samples was 24 μg/kg (2012) and 276 μg/kg (2013). Negligible amount, or absence, of DON was detected all over the trials during the two cropping years with the exception of the wetter and colder field site A, especially in 2013. In this site, the highest average values of DON were achieved both in the tilled (414 μg/kg) and in minimum-tillage management (92 μg/kg). However, data collected about the levels of DON contamination were all very far from the maximum limit (1750 μg/kg) set for unprocessed durum wheat (EC Regulation 1881/2006 and 1126/2007). These results were in agreement with the meteorological data collected all over the 2-year period that showed a rainy condition in the year 2013 generally higher than in 2012. The suitable conditions for an outbreak of FHB in field and DON production, such as rainfall occurrence and high percentage of relative humidity, raised from meteorological mean data collected. By all data collected, a slight positive—but highly significant—correlation (*p* < 0.001) was found between the parameters DON and fungal DNA detected in kernels. Several studies reported positive correlations between disease incidence and mycotoxin accumulation (Burlakoti et al., 2007); however this question might be more complex due to the not always positive and significant relationship between wheat varieties with high FHB resistance and low levels of DON detected (Boutigny et al., 2008).

Regarding the quality aspects assessed with protein and gluten content, alveographic W, and alveographic (P/L) ratio, our analysis confirmed the effects of the environment and cultivar type on key technological parameters for the quality level of the end-product (pasta; Mariani et al., 1995; Raciti et al., 2005). The protein content in durum wheat is influenced by environment parameters, with high temperature and water shortage that can significantly affect both the content and protein composition in Mediterranean climate (Flagella, 2006). The negative correlation between protein content and yield parameters was in agreement with previous data (Mangini, 2006; Blanco et al., 2011). These results confirm the importance of the genotype-environment interaction in determining the protein content particularly influenced by additive effects of environment (Mariani et al., 1995). Apparently, this interaction has scarce effect on gluten index parameter (Ames et al., 1999) which however is significantly affected by the temperature trend during grain filling (Flagella et al., 2010; Fois et al., 2011).

In the same way the year and site of cultivation had a significant “weight” (*p* < 0.001) on the brown index, a negative quality trait subjected also to the environmental effect. As expected the yellow index was influenced (*p* < 0.001) by environmental factor (year) but also the role of genotype influenced was confirmed by the significant effect (*p* < 0.001) of cultivar parameter. However, no significant differences was found by the overall judgment of the end-product (pasta) probably due to the minimal differences of mean values within the study sites.

## Conclusions

This study suggested that also in Southern Italy, a growing area under Mediterranean climate suitable for durum wheat cropping, weather conditions, and soil management may affect not only several quality traits of durum wheat, but also infection by trichothecene-producing *Fusarium* and accumulation of DON in kernels. More in general, differences emerging among the sites and the cultivars in relation to fungal growth and DON content (even if both kept at low level) apparently suggest how these parameters can be finely regulated by environmental parameters at micro-scale.

Mediterranean climatic conditions are mostly unsuitable for outbreak of FHB and field contamination by DON. This likely occurs because of the scarcity of rainfall events and amount during the anthesis. However, FHB outbreak can still occur particularly in moist years in production areas located at “high” elevation. Under such circumstances, the wetter and colder conditions promote infection and disease spreading also in suitable growing areas.

In conclusion, weather parameters, especially rainfall and air humidity with low values of temperature appear the main drivers to settle conductive or non-conductive conditions for TR-producing *Fusarium* spp. development, although the choice of type of tillage and the selection of suitable cultivars can significantly affect the level of disease spreading.

## Author contributions

VS: conceived of the study, participated in its design and coordination, drafts, and revises critically the manuscript. Conception and design of the nucleic acid extraction and Real Time PCR methods, Fusarium spp. infection and FHB index; acquisition, analysis, interpretation of data for the work; GA: contributes to the conception or design of the work. Acquisition, analysis, or interpretation of data for the mycotoxins analysis and yield and grain quality. Drafting the work and revising it critically for important intellectual content; GC: contributions to the acquisition, analysis, or interpretation of data for the work. Drafting and the work or revising it critically; GI: contributions to data analysis, interpretation of data for the work, Drafting the work; CF: Drafting the work, revising it critically for important intellectual content, conceived of the study, participated in its design and coordination; FS: Drafting the work, revising it critically for important intellectual content, conceived of the study, participated in its design and coordination; MR: conceived of the study, participated in its design and coordination, drafts and revises critically the manuscript. Conception and design of the nucleic acid analysis, Fusarium spp. infection and FHB index; acquisition, analysis, interpretation of data for the work; GA: contributes.

## Funding

The present study was funded by the MiPAF through the project “VAFRUMIC” within the OIGA (DM 18829/2009)—“Progetti Ricerca e Sviluppo” framework and by Progetti Ateneo Sapienza University of Rome (Year 2014—prot. C26A14AFZ7).

### Conflict of interest statement

The authors declare that the research was conducted in the absence of any commercial or financial relationships that could be construed as a potential conflict of interest. The handling editor declared an intended collaboration with the author MR, but states that the process nevertheless met the standards of a fair and objective review.
