# Supplementary material for: Climate, Soil Management, and Cultivar Affect Fusarium Head Blight Incidence and Deoxynivalenol Accumulation in Durum Wheat of Southern Italy
Source: Front Microbiol. 2016 Jun 30;7:1014. doi: 10.3389/fmicb.2016.01014 (PMC4928167; doi:10.3389/fmicb.2016.01014)
Supplement: Supplementary file 2 [file DataSheet2.docx]

**Table S1**. Properties of the topsoil (0-30 cm) at the three study sites. For each parameter, data refer to mean ± standard deviation. Different letters indicate statistically significant differences (Duncan test; *P* < 0.05).

|  | **Study site** | | |
| --- | --- | --- | --- |
| **Soil parameters** | **A** | **B** | **C** |
| pH | 8.17 ± 0.32 a | 8.35 ± 0.26 a | 8.42 ± 0.45 a |
| Electrical conductivity (µS cm^-1^) | 91.53 ± 12.41 b | 136.21 ± 15.21 a | 124.53 ± 9.28 a |
| Organic C (g kg^-1^) | 28.33 ± 3.16 a | 26.94 ± 2.21 a | 25.27 ± 3.37 a |
| Total N (g kg^-1^) | 2.36 ± 0.25 ab | 1.94 ± 0.33 b | 2.52 ± 0.47 a |
| C / N ratio | 12.01 ± 3.77 a | 13.88 ± 3.01 a | 10.03 ± 3.59 a |
| Available P_2_O_5_ (mg kg^-1^) | 18.39 ± 4.83 a | 23.21 ± 6.21 a | 16.08 ± 4.70 a |
| K^+^ (cmol_(+)_ kg^-1^) | 1.01 ± 0.12 a | 1.22 ± 0.19 a | 1.13 ± 0.28 a |

**Table S2**. Effect of year (2012 and 2013), soil management regime (till *vs* no-till), study site along a a gradient of decreasing rainfall (A= Pauroso, B = Diga, C = Serrone), and cultivar (Claudio, Normanno, Simeto, Svevo) on wheat quality assessed by seven parameters. Different letters indicate statistically significant differences among different levels for each experimental factor (post-hoc Duncan test from GLM in Table 1, *P*<0.05).

| Factor | Level | Gluten (%) | Gluten index | (W, J x10 ^-4^) | P/L ratio | Yellow index | Brown index | Sensorial assessment overall score |
| --- | --- | --- | --- | --- | --- | --- | --- | --- |
| Year | 2012 | 9.02b | 86.25a | 205.03b | 2.14b | 25.53a | 11.32b | 53.80a |
|  | 2013 | 10.53a | 86.47a | 287.29a | 3.57a | 23.04b | 12.23a | 53.58a |
| Soil management | no-till | 9.63a | 87.13a | 238.04a | 2.89a | 24.29a | 11.94a | 52.97a |
|  | till | 9.92a | 85.59a | 254.25a | 2.82a | 24.28a | 11.62b | 54.41a |
| Study site | A | 10.70a | 88.67a | 283.12a | 4.02a | 24.77a | 12.35a | 54.22a |
|  | B | 9.15b | 81.24b | 210.68c | 2.30b | 24.04a | 11.42b | 54.14a |
|  | C | 9.48b | 89.17a | 244.62b | 2.24b | 24.06a | 11.57b | 52.70a |
| Cultivar | Claudio | 9.45b | 92.63a | 228.33a | 1.93c | 22.75b | 11.20b | 53.47a |
|  | Normanno | 8.87b | 95.86a | 254.16a | 2.91b | 26.57a | 12.19a | 52.27a |
|  | Simeto | 10.06a | 79.54b | 238.33a | 4.14a | 21.96b | 12.27a | 55.05a |
|  | Svevo | 10.71a | 77.41b | 263.75a | 2.44bc | 25.85a | 11.46b | 53.97a |
|  |  |  |  |  |  |  |  |  |

**Table S3.** Incidence of infected kernels (%) in 2012 and 2013 and incidence of kernels infected by *Fusarium* spp.*, Aspergillus* spp. and *Penicillium* spp.

|  | Incidence of infected kernels (%) | |
| --- | --- | --- |
|  | 2012 | 2013 |
| All fungi | 13,2 ± 4,7 | 86,0 ± 5,5 |
| *Fusarium* spp. | 13±7.2 | 86±8.7 |
| *Aspergillus* spp. | 11±6.3 | 67±6.9 |
| *Penicillium* spp. | 3±2.8 | 8±3.5 |

**Table S4**. Cross-correlation matrix between wheat production, kernel infection of TR-producing *Fusarium*, deoxynivalenol toxin (DON) accumulation in kernel and several dough quality parameters. Signiﬁcant correlations from regression analysis (P < 0.05) are in bold.

|  | **1** | **2** | **3** | **4** | **5** | **6** | **7** | **8** | **9** | **10** |
| --- | --- | --- | --- | --- | --- | --- | --- | --- | --- | --- |
| **ng DNA *Fusarium* g^-1^ (11)** | -0,15 | **0,50** | 0,04 | -0,08 | **0,58** | 0,28 | 0,25 | **0,46** | **0,63** | **-0,30** |
| **Yield (t/ha) (10)** | -0,08 | **-0,71** | 0,19 | -0,05 | **-0,38** | -0,10 | **-0,52** | **-0,67** | **-0,45** |  |
| **DON (ppb) (9)** | -0,05 | **0,52** | -0,10 | -0,10 | **0,57** | 0,18 | **0,38** | **0,52** |  |  |
| **Protein (%) (8)** | 0,19 | **0,74** | -0,24 | 0,15 | **0,74** | -0,08 | **0,84** |  |  |  |
| **Gluten (%) (7)** | 0,22 | **0,45** | -0,20 | -0,04 | **0,66** | **-0,35** |  |  |  |  |
| **Gluten index (6)** | -0,02 | 0,11 | 0,10 | -0,21 | 0,23 |  |  |  |  |  |
| **W (J x10^-4^) (5)** | 0,15 | **0,45** | -0,08 | -0,10 |  |  |  |  |  |  |
| **P/L (4)** | 0,04 | 0,21 | **-0,40** |  |  |  |  |  |  |  |
| **Yellow index (3)** | -0,13 | -0,18 |  |  |  |  |  |  |  |  |
| **Brown index (2)** | 0,10 |  |  |  |  |  |  |  |  |  |
| **Sensorial assessment score (1)** |  |  |  |  |  |  |  |  |  |  |

**Figure S1.** Location of the study area (red dot in top-left panel) in Southern Italy (under the red line boundary) and pictures of the three study sites with meteorological stations (Site A: 41°02’00.99’’N, 15°27’10.82’’E, 827 m a.s.l.; Site B: 41°01’20.65’’N, 15°30’05.55’’E, 520 m a.s.l.; Site C: 41°03’16.98’’N, 15°30’32.45’’E, 513 m a.s.l.).


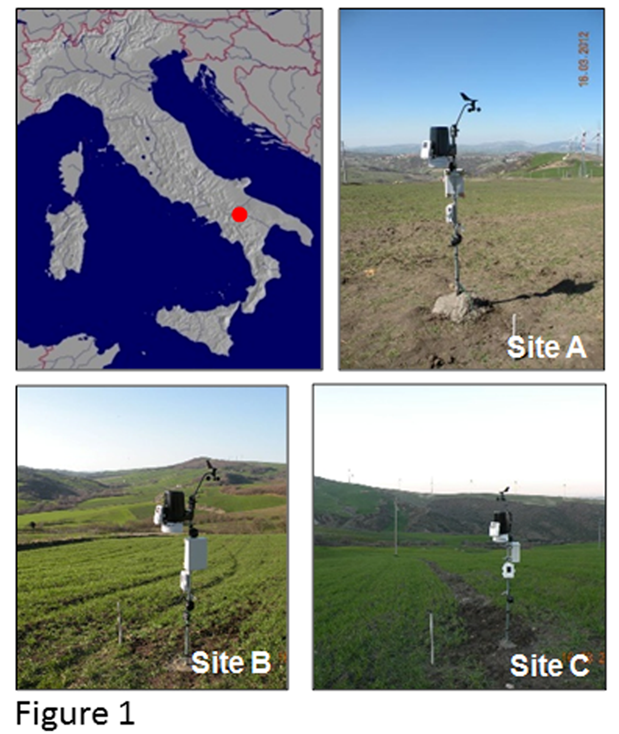


**Figure S2**. Average monthly temperature, relative humidity and cumulated rainfall in May 2012 and May 2013 at the three study sites (A, B, and C).

**Figure S3**. Climatic conditions recorded from 1 to 31 May 2012 at the three study sites (A, B, and C). From top to bottom, data refer to air temperature, relative humidity and rainfall, respectively.

**Figure S4**. Climatic conditions recorded from 1 to 31 May 2013 at the three study sites (A, B, and C). From top to bottom, data refer to air temperature, relative humidity and rainfall, respectively.
